# Galcanezumab in patients with episodic migraine: results from the open-label period of the phase 3 PERSIST study

**DOI:** 10.1186/s10194-023-01613-1

**Published:** 2023-08-04

**Authors:** Jiying Zhou, Lianmei Zhong, Debashish Chowdhury, Kirill Skorobogatykh, Guogang Luo, Xiaosu Yang, Mingjie Zhang, Lingli Sun, Hui Liu, Chenxi Qian, Shengyuan Yu

**Affiliations:** 1 Department of Neurology, The First Affiliated Hospital of Chongqing Medical University, Chongqing, China; 2 Department of Neurology, First Affiliated Hospital of Kunming Medical University, Kunming, China; 3 Gobind Ballabh Pant Institute of Post Graduate Medical Education and Research, New Delhi, India; 4 University Headache Clinic, Moscow, Russia; 5 Stroke Centre and Department of Neurology, The First Affiliated Hospital of Xi’an Jiaotong University, Xi’an, China; 6 Department of Neurology, Xiangya Hospital, Central South University, Changsha, China; 7 Department of Neurology, Chinese PLA General Hospital, Beijing, China; 8 Eli Lilly and Company, Shanghai, China

**Keywords:** Calcitonin gene-related peptide, Galcanezumab, Humanized monoclonal antibody, Episodic migraine”

## Abstract

**Background:**

The phase 3 randomized PERSIST study demonstrated the efficacy and tolerability of galcanezumab, a humanized anti-calcitonin gene-related peptide (CGRP) monoclonal antibody for prevention of episodic migraines. We present findings from the open-label extension (OLE) of PERSIST, which evaluated the long-term efficacy and safety of galcanezumab in patients from China, India, and Russia.

**Methods:**

Patients completing the 3-month double-blind period of PERSIST were eligible for the 3-month OLE. Patients previously randomized to galcanezumab (GMB/GMB group) continued to receive galcanezumab 120 mg at all three visits during the OLE whereas patients randomized to placebo received a 240 mg loading dose of galcanezumab and then two 120 mg doses (PBO/GMB group). The primary outcome was the mean change (from double-blind baseline) in the number of monthly migraine headache days (MHDs) to month 6. Other endpoints included percent reduction in monthly MHDs from double-blind baseline to month 6, functional outcomes, safety and tolerability.

**Results:**

Overall, 99% of patients completing the double-blind period entered the OLE, and 96% completed through month 6. Patients in the GMB/GMB group achieved continued improvements in efficacy, with the reduction from baseline in the mean number of monthly MHDs, and slightly increasing from 4.01 days at the end of the double-blind period to 4.62 at the end of the OLE. Of patients who were ≥ 50% responders to galcanezumab at month 3, 66% maintained this response through to month 6. Patients in the PBO/GMB group experienced a rapid reduction in the number of monthly MHDs after initiation of galcanezumab, with a mean reduction from baseline of 4.56 days by month 6. The long-term benefits of galcanezumab were also supported by improvements in other efficacy and functional endpoints. All safety findings were consistent with the known long-term safety profile of galcanezumab; no patients experienced a treatment-related serious adverse event.

**Conclusions:**

Galcanezumab was efficacious and well-tolerated in patients with episodic migraine from China, India and Russia, for up to 6 months.

**Trial registration:**

ClinicalTrisABSTRACT_pals.gov NCT03963232, registered May 24, 2019.

**Supplementary Information:**

The online version contains supplementary material available at 10.1186/s10194-023-01613-1.

## Background

Migraine was estimated to affect more than one billion individuals worldwide in 2019 [1], with an age-standardized point prevalence of 14.1% globally and 11.7% in China [1]. Migraine is an important cause of disability worldwide and the number of years lived with disability due to migraine globally was 42.1 million in 2019. For Central, East, Southeast and South Asia, the number of years lived with a disability due to migraine was estimated to be 0.5 million, 7.3 million, 4.2 million and 9.8 million, respectively [2].

Among individuals affected by migraines, around one-third (34–39%) may be candidates for preventive therapy [3, 4]; however, most current preventive therapies were initially developed for other therapeutic uses. Therefore, these therapies may have low adherence and persistence due to poor efficacy and tolerability [5, 6].

Calcitonin gene-related peptide (CGRP) has a key pathophysiological role in migraine and is expressed widely in the central and peripheral nociceptive system [7], representing a novel therapeutic target for prevention of episodic migraine. A number of monoclonal antibodies against CGRP or its receptor have been shown to provide a preventive effect for migraine and may have an improved benefit-risk profile versus historical preventive treatments [8, 9].

Galcanezumab has been assessed in multiple phase 3 placebo-controlled trials for episodic, chronic, and treatment resistant migraine [10–15]. The phase 3, randomized, double-blind, placebo-controlled PERSIST study in China, India, and Russia randomized 520 patients and evaluated galcanezumab 120 mg in patients with episodic migraine [16]. Findings from the 3-month double-blind period demonstrated that galcanezumab 120 mg resulted in significantly higher overall mean reductions in migraine headache days (MHDs) per month compared with placebo; by 3.81 days versus 1.99 days (*p* < 0.0001) [16]. Galcanezumab was also associated with acceptable tolerability, with low rates of serious adverse events (SAEs) and few discontinuations due to treatment-emergent adverse events (TEAEs). After completion of the double-blind period of the PERSIST trial, patients could enter a 3-month open-label extension (OLE) period. Here, we report results of the OLE study, which further evaluated the efficacy and safety of galcanezumab in patients with episodic migraine from China, India and Russia up to 6 months.

## Methods

### Study design and treatment

The phase 3 PERSIST study (NCT03963232) was conducted at 26 centers in China, 20 in India, and 4 in Russia (40 total). There were five study periods (Suppl. Figure 1): initial screening and washout; a prospective baseline period; a 3-month, randomized, double-blind, placebo-controlled treatment period; a 3-month OLE; and a 4-month post-treatment phase. As previously reported in detail [16], during the double-blind treatment period, patients were randomized (1:1) to galcanezumab 120 mg (as a monthly subcutaneous injection with a 240 mg loading dose) or matching placebo. All patients who completed the double-blind treatment period could enter the OLE and receive open-label study drug. Patients from the prior placebo group received a 240 mg loading dose of galcanezumab at Visit 7 and subsequently 120 mg at Visits 8 and 9. Patients from the prior galcanezumab group continued to receive galcanezumab 120 mg at all three visits during the open-label period. Sites and patients remained blinded to patients’ previous treatment assignment. To preserve blinding at Visit 7, all patients received two injections: the prior placebo group received two injections of galcanezumab 120 mg and the prior galcanezumab group received one injection of galcanezumab 120 mg and one injection of placebo.

### Patients

Eligibility criteria for the PERSIST trial have been described previously [16]. In brief, the study included adults (18–65 years) with episodic migraine [17].

### Assessments and endpoints

The primary efficacy measure was mean change in the number of monthly MHDs from double-blind baseline (the prospective baseline period) to month 6. Secondary endpoints included response rates (based on percent reduction in monthly MHDs from double-blind baseline to month 6), functional outcomes, safety and tolerability.

The mean change in monthly MHDs was derived from the ePRO system, in which patients recorded information about headaches (including medication used) and migraine-associated symptoms each day. Response rates were then estimated as the percentage of patients with reductions of ≥ 50%, ≥ 75% and 100% in monthly MHDs from double-blind baseline. A maintained ≥ 50% response was defined as a ≥ 50% reduction in monthly MHDs from baseline to month 3 that was maintained throughout the OLE. Functional outcomes were assessed as described previously [16] using the Migraine-Specific Quality of Life Questionnaire (MSQ) [18], the Patient Global Impression of Severity (PGI-S) scale [19], and the Migraine Disability Assessment (MIDAS) score [20]. The MSQ was conducted at randomization and monthly until month 6, whereas the PGI-S and MIDAS score questionnaires were administered at baseline, month 3 (end of double-blind period) and month 6 (end of OLE).

Safety was assessed by monitoring TEAEs, SAEs, deaths, adverse events leading to discontinuation, laboratory tests, electrocardiograms, vital signs, and body weight. Levels of antidrug antibodies (ADA) and neutralizing ADAs were measured to assess immunogenicity. Treatment-emergent ADAs (TE-ADAs) were defined as a negative baseline ADA result followed by a positive post-baseline ADA result with a titer ≥ 1:20 (treatment-induced) or positive baseline and post-baseline ADA results with a ≥ fourfold increase in titer (treatment-boosted ADA).

### Statistical analysis

Efficacy and safety analyses were performed in all randomized patients who received at least one dose of the study drug in groups defined by treatment assignment during the double-blind period, i.e., patients previously randomized to galcanezumab (GMB/GMB group) or to placebo (PBO/GMB group). The efficacy analysis included data from both the double-blind and OLE periods. Patients who have a baseline (from the double-blind phase) and at least one post-baseline observation were included in the analysis. Continuous efficacy endpoints were analyzed using a restricted maximum likelihood-based mixed model for repeated measures (MMRM). Binary efficacy endpoints with repeated measurements were analyzed with a categorical, pseudo-likelihood-based repeated measures analysis implemented using a generalized linear mixed model procedure (GLIMMIX). Except for the efficacy analyses on MHDs or categorical analysis of response rate (such as 50% response rate) derived from MHDs, in which the continuous value of baseline MHDs was used as a covariate, all other efficacy analyses included baseline number of MHDs category (< 8 vs ≥ 8) as a covariate in the MMRM and GLIMMIX model.

Comparisons were claimed to be statistically significant if two-sided *p*-values were less than 0.05. TEAEs were summarized descriptively. Immunogenicity was assessed in all patients who received galcanezumab. All statistical analyses were performed using SAS version 9.4 or higher.

## Results

### Patient disposition and demographics

**Fig. 1:**
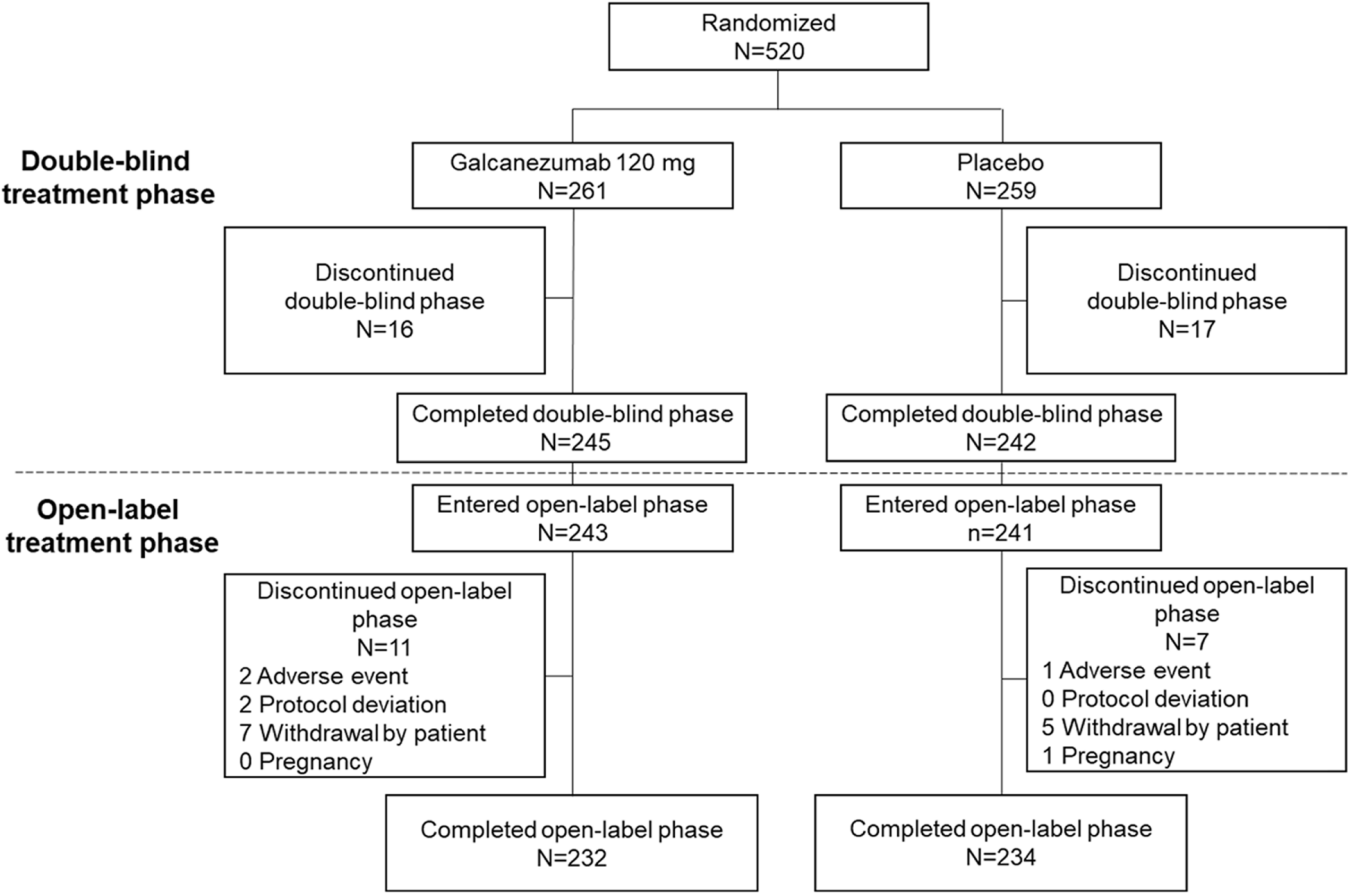
Patient disposition

Almost all of the 487 patients who completed double-blind treatment (*N* = 484, 99.4%) entered the OLE (Fig. 1). This included 243 and 241 patients previously randomized to galcanezumab (GMB/GMB) or placebo (PBO/GMB), respectively. The majority of patients (466/484, 96.3%) completed the OLE: 95.5% in the GMB/GMB and 97.1% in the PBO/GMB group. The mean duration of exposure to treatment was 92.2 days during the OLE and mean treatment compliance was > 97%. In total, 18 patients discontinued treatment during the OLE; three due to an adverse event, two due to a protocol deviation, 12 withdrew from the study, and one due to pregnancy.

Baseline demographics and disease characteristics for the patients who enrolled in the OLE are presented in Table 1; the majority were female (73.3%) and from China (76.9%), with a mean age of 36.9 (standard deviation [SD]: 9.6) years and a mean migraine illness duration of 12.6 (SD: 8.6) years. At baseline, the mean monthly MHDs was 8.25 days and about half (55.4%) were experiencing ≥ 8 monthly MHDs, indicating a balance of low- and high-frequency MHD patients. Patients typically had moderately impaired daily functioning (mean MSQ-Role Function Restrictive score of 56.1), very severe disability (mean total MIDAS score of 46.9), and had moderate migraine severity (mean PGI-S score of 4.4).

**Table 1:** Demographics and baseline disease characteristics in open label extension period

	**GMB/GMB (*N* = 243)**	**PBO/GMB (*N* = 241)**
Age, years	37.2 (9.3)	36.5 (9.8)
Females, n (%)	175 (72.0)	180 (74.7)
Race, n (%)		
Asian	223 (91.8)	222 (92.1)
White	20 (8.2)	19 (7.9)
Body mass index, kg/m^2^	23.4 (3.7)	22.5 (3.1)
Region		
China	185 (76.1)	187 (77.6)
India	38 (15.6)	35 (14.5)
Russia	20 (8.2)	19 (7.9)
MHDs per month	8.2 (2.8)	8.3 (2.7)
MHDs with acute medication use per month	5.3 (5.0)	5.0 (4.5)
Migraine frequency, n (%)		
< 8 days/month	110 (45.3)	106 (44.0)
≥ 8 days/month	133 (54.7)	135 (56.0)
Duration of migraine illness, years	12.7 (9.1)	12.4 (8.2)
ICHD MHDs per month	6.3 (3.3)	6.2 (3.2)
Headache days per month	9.1 (3.3)	9.1 (2.9)
Migraine attacks per month	5.5 (1.8)	5.6 (1.7)
MSQ score		
Total	61.0 (15.9)	62.1 (15.3)
Role Function-Restrictive	55.3 (15.4)	56.9 (14.8)
Role Function-Preventive	65.6 (18.6)	66.1 (17.5)
Emotional Function	68.2 (22.2)	68.7 (21.7)
PGI-S	4.5 (1.3)	4.4 (1.3)
MIDAS total score	48.8 (37.4)	45.0 (35.0)

Data are presented as mean (standard deviation) unless otherwise specified

*GMB* Galcanezumab, *ICHD* International Classification of Headache Disorders, *MHD* Migraine headache day, *MIDAS* Migraine Disability Assessment, *MSQ* Migraine Specific Quality of Life Questionnaire, *N* Number of patients in the analysis population, *n* Number of patients within each specific category, *PBO* Placebo, *PGI-S* Patient Global Impression of Severity

### Efficacy

**Fig. 2:**
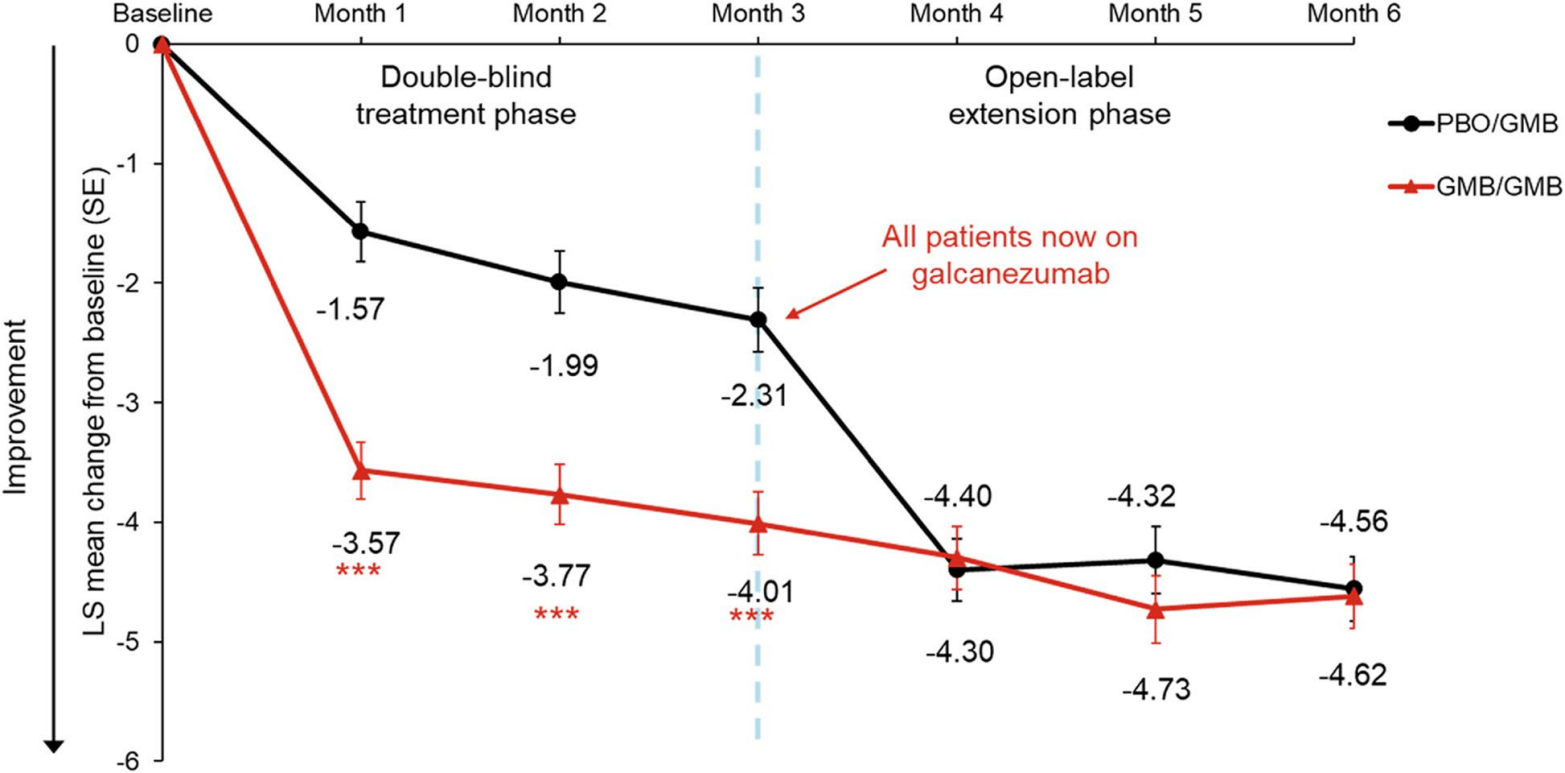
LS mean change from baseline in monthly migraine headache days through to Month 6 GMB, galcanezumab; LS, least squares; PBO, placebo; SE, standard error ***p < 0.0001 (comparison between galcanezumab and placebo treatment groups)

At the end of the OLE (month 6), patients in the PBO/GMB and GMB/GMB groups had a least squares (LS) mean reduction from baseline in monthly MHDs of 4.56 and. 4.62, respectively (Table 2; Fig. 2). In the GMB/GMB group, patients demonstrated sustained improvements throughout the OLE, with the LS mean reduction in the number of monthly MHDs from baseline increasing from 4.01 days at the end of the double-blind period to 4.62 at the end of the OLE (Fig. 2). In the PBO/GMB group, patients experienced a fast decrease in the number of monthly MHDs after initiating galcanezumab treatment, reaching that achieved by the GMB/GMB group by month 4, and subsequently maintaining the reduction (Fig. 2).

**Table 2:** Efficacy and quality of life endpoints

Endpoint	Time	Change from baseline^a^			Treatment difference	
		**GMB/GMB**	**PBO/GMB**	**LS Mean (95% CI)**^**b**^		***p*-value**
		**LS Mean (SE)**	**LS Mean (SE)**			
Monthly MHDs	Month 3	-4.01 (0.26)	-2.31 (0.27)	-1.70 (-2.34, -1.06)		< 0.0001
	Month 6	-4.62 (0.27)	-4.56 (0.27)	-0.06 (-0.72, 0.60)		0.8607
≥ 50% response rate	Month 3	59.7 (3.1)^c^	35.9 (3.1)^c^	2.64 (1.83, 3.80)		< 0.0001
	Month 6	70.9 (3.0)^c^	67.2 (3.1)^c^	1.19 (0.80, 1.76)		0.3906
≥ 75% response rate	Month 3	28.3 (2.9)^c^	18.4 (2.5)^c^	1.75 (1.14, 2.70)		0.0108
	Month 6	46.1 (3.3)^c^	47.1 (3.3)^c^	0.96 (0.67, 1.38)		0.8177
100% response rate	Month 3	13.4 (2.3)^c^	6.2 (1.6)^c^	2.34 (1.21, 4.52)		0.0114
	Month 6	21.5 (2.7)^c^	23.1 (2.8)^c^	0.91 (0.59, 1.41)		0.6815
Monthly MHDs treated with acute medication	Month 3	-2.64 (0.25)	-0.77 (0.25)	-1.86 (-2.46, -1.27)		< 0.0001
	Month 6	-2.82 (0.24)	-2.40 (0.24)	-0.43 (-0.99, 0.13)		0.1330
MSQ-RFR	Month 3	20.71 (0.99)	15.27 (1.01)	5.44 (3.03, 7.86)		< 0.0001
	Month 6	23.72 (1.02)	24.92 (1.04)	-1.20 (-3.70, 1.31)		0.3473
PGI-S	Month 3	-0.90 (0.09)	-0.70 (0.09)	-0.20 (-0.41, 0.01)		0.0559
	Month 6	-1.18 (0.10)	-1.09 (0.10)	-0.09 (-0.32, 0.15)		0.4701
MIDAS Total Score	Month 3	-23.09 (2.72)	-11.12 (2.80)	-11.97 (-18.53, -5.42)		0.0004
	Month 6	-27.77 (2.39)	-27.24 (2.48)	-0.54 (-5.97, 4.90)		0.8459

^a^All values reported as change from baseline with the exception of response rates, which are absolute values at the timepoint indicated

^b^All values reported as LS square mean difference, with the exception of response rates, which are reported as odds ratios

^c^Model estimated rate

*CI* Confidence interval, *GMB* Galcanezumab, *LS* Least squares, *MHD* Migraine headache day, *MIDAS* Migraine Disability Assessment, *MSQ-RFR* Migraine Specific Quality of Life Questionnaire Role Function-Restrictive, *NA* Not applicable, *PBO* Placebo, *PGI-S* Patient Global Impression of Severity, *SE* Standard error

**Fig. 3:**
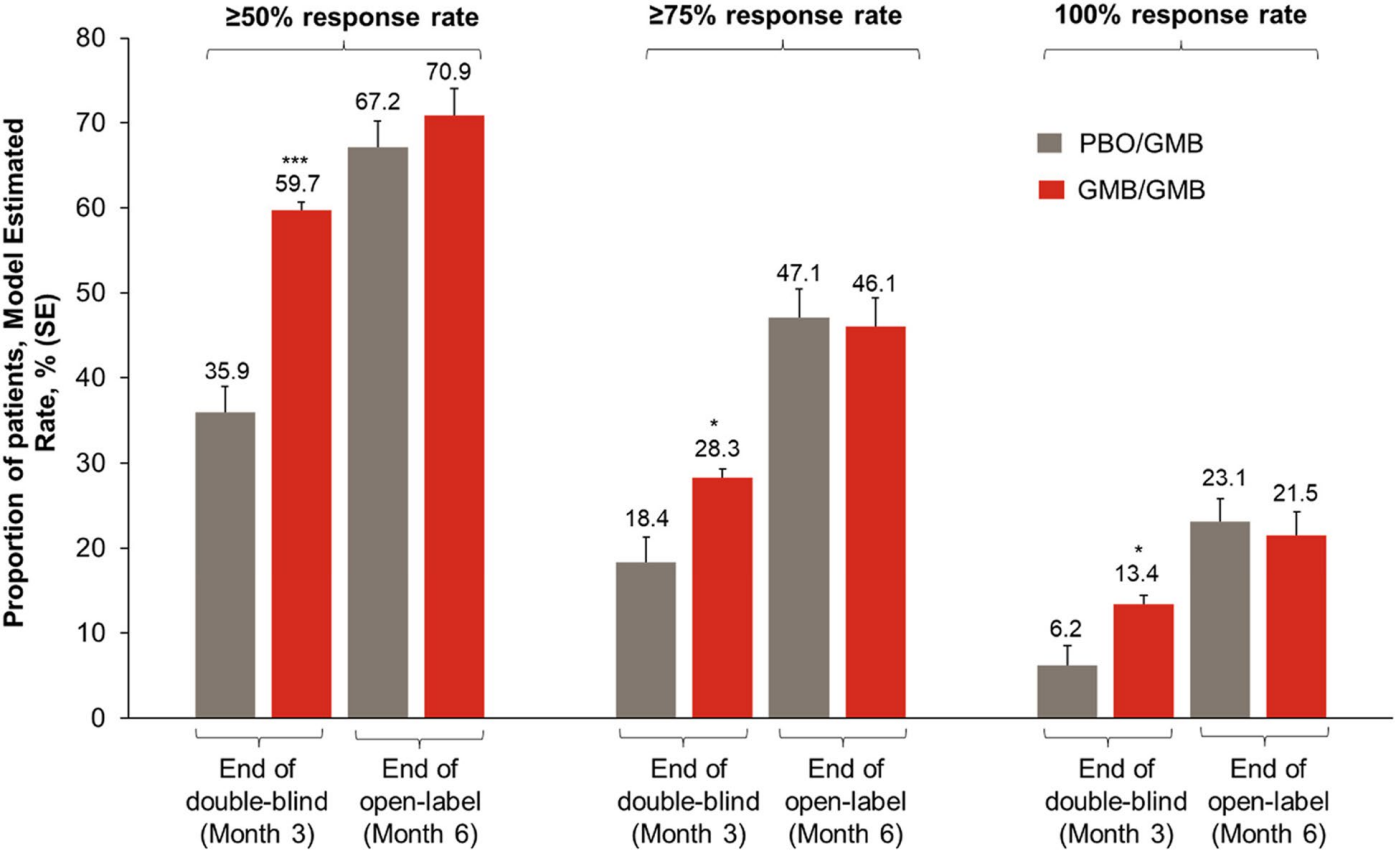
Percentage of patients with ≥ 50%, ≥ 75%, and 100% reductions in monthly migraine headache days at month 3 (end of double-blind period) and month 6 (end of open-label period) DB, double-blind; GMB, galcanezumab; OL, open-label; PBO, placebo; SE, standard error *p < 0.05 versus placebo; ***p < 0.0001 versus placebo

Response rates in patients in the GMB/GMB group increased from the end of the double-blind period to the end of the OLE. The proportion of patients achieving a ≥ 50% response increased from 59.7% to 70.9%, the proportion achieving a ≥ 75% response increased from 28.3% to 46.1%. and the proportion achieving a 100% response increased from 13.4% to 21.5% (Table 2, Fig. 3). After starting treatment with open-label galcanezumab, the percentage of patients in the PBO/GMB group achieving all three thresholds of response had increased by month 6.

Overall, 142 patients who were randomized to the galcanezumab group and were ≥ 50% responders at month 3 continued into the OLE. Of these, 66.2% (94/142) maintained a ≥ 50% response through to month 6. At the end of the OLE (month 6), patients in the GMB/GMB group had a further mean reduction in the number of monthly MHDs with acute headache medication; a reduction was also observed for the PBO/GMB group. Mean changes in the functional endpoints (MSQ-RFR, MIDAS, PGI-S) showed a similar pattern, indicating that further improvements were observed in the GMB/GMB group at month 6, and a rapid improvement occurred for patients in the PBO/GMB group (Table 2). MSQ-RFR domain scores increased by 23.72 following 6 months of treatment with galcanezumab, indicating a change from “moderately impaired” to “mildly impaired” performance of daily activities limited by migraine.

### Safety

During the open-label period, TEAEs were reported by 94 (38.7%) and 83 (34.4%) patients in the GMB/GMB and PBO/GMB groups, respectively (Table 3). Most TEAEs (96.0%) were reported to be either mild or moderate in severity. For patients in the GMB/GMB group, the most common TEAEs during the open-label period were injection site reaction, injection site pruritus and upper respiratory tract infection, affecting 4.9%, 3.3% and 2.9% of patients, respectively. For patients in the PBO/GMB group, the most common TEAEs during the open-label period were injection site pain, upper respiratory tract infection and injection site erythema, affecting 2.5%, 2.5% and 2.1% of patients, respectively (Table 3). Fourteen SAEs were reported by 12 patients in total during the OLE. Three patients in the GMB/GMB group reported four SAEs (intestinal obstruction, mucosal infection, traumatic ulcer, and uterine polyp). Nine patients in the PBO/GMB group reported ten SAEs (borderline personality disorder, COVID-19 pneumonia, carpal tunnel syndrome, ectopic pregnancy, hemorrhoids, ligament sprain, limb injury, migraine, pain in extremity, and tension headache) and no SAEs were considered related to study treatment by the investigator. No patients died during the total study period. TEAEs leading to treatment discontinuation during the OLE occurred in two patients in the GMB/GMB group (abdominal discomfort and iritis) and one patient in the PBO/GMB group (an SAE of COVID-19 pneumonia).

**Table 3:** Overview of adverse events in the open-label treatment phase

Preferred term	GMB/GMB (*N* = 243) n (%)	PBO/GMB (*N* = 241) n (%)	Total (*N* = 484) n (%)
Deaths	0	0	0
Patients with ≥ 1 SAEs	3 (1.2)	9 (3.7)	12 (2.5)
Patients discontinuing due to an AE	2 (0.8)	1 (0.4)	3 (0.6)
Patients with ≥ 1 TEAE	94 (38.7)	83 (34.4)	177 (36.6)
Patients with ≥ 1 TEAE relating to treatment	33 (13.6)	23 (9.5)	56 (11.6)
TEAEs^a^			
Injection site reaction	12 (4.9)	4 (1.7)	16 (3.3)
Injection site pruritis	8 (3.3)	4 (1.7)	12 (2.5)
Upper respiratory tract infection	7 (2.9)	6 (2.5)	13 (2.7)
Nasopharyngitis	6 (2.5)	2 (0.8)	8 (1.7)
Injection site erythema	4 (1.6)	5 (2.1)	9 (1.9)
Protein urine present	4 (1.6)	3 (1.2)	7 (1.4)
Pyrexia	4 (1.6)	3 (1.2)	7 (1.4)
Abdominal pain upper	4 (1.6)	1 (0.4)	5 (1.0)
Weight increased	4 (1.6)	1 (0.4)	5 (1.0)
Anemia	4 (1.6)	0	4 (0.8)
Abdominal discomfort	1 (0.4)	4 (1.7)	5 (1.0)
Injection site discomfort	1 (0.4)	4 (1.7)	5 (1.0)
Injection site pain	0	6 (2.5)	6 (1.2)

^a^TEAEs occurring in ≥ 1.5% of patients in any open-label treatment group

*GMB* Galcanezumab, *N* Number of patients in the analysis population, *n* Number of patients within each specific category, *PBO* Placebo, *SAE* Serious adverse event, *TEAE* Treatment-emergent adverse event

### Immunogenicity

Among 482 evaluable patients treated with galcanezumab during the double-blind and/or OLE period, 71 (14.7%) had ADAs present at baseline, with 37 patients (7.7%) having neutralizing ADAs. TE-ADAs were detected during galcanezumab treatment in 63 (13.1%) patients, including 59 patients (12.2%) who developed neutralizing ADAs. During the entire study, including the 4-month post-treatment period, 188 galcanezumab-treated patients developed TE-ADAs, including 186 patients (37.6%) who developed neutralizing ADAs. No meaningful relationship was observed between ADAs and efficacy or tolerability of galcanezumab.

## Discussion

In this 3-month OLE of the PERSIST study, galcanezumab 120 mg continued to be efficacious in patients from China, India and Russia with episodic migraine for up to 6 months, with a generally good safety profile.

The findings in predominantly Asian patients are consistent with published results from the OLE of the phase 3 CONQUER episodic migraine populations [15]. Although CONQUER study enrolled both chronic and episodic migraine populations, we compared here only the CONQUER episodic migraine population with the PERSIST study, and these indirect comparisons must be interpreted with caution. It is interesting to note that the efficacy of galcanezumab at the end of the open-label phase of the present study appeared to be higher compared with results from the predominantly Caucasian population with episodic migraine enrolled in CONQUER (reduction in monthly MHDs from baseline to Month 6: 4.6 vs 3.8; percentage achieving a ≥ 50% response rate at Month 6: 70.9% vs 57.3%) [15]. Furthermore, there appeared to be a greater placebo effect in PERSIST, with 35.9% of placebo-treated patients achieving a ≥ 50% response at the end of the double-blind phase compared with 20.8% of placebo-treated patients in CONQUER episodic migraine populations. However, other differences between the PERSIST and CONQUER episodic migraine populations should be noted, including a lower mean age (GMB/GMB 37.2/ PBO/GMB 36.5 years vs. 45.9/46.3 years), less mean monthly MHDs (8.2/8.3 days vs. 9.5/9.2 days), and a shorter mean duration of migraine illness (12.7/12.4 years vs. 21.7/22.9 years) at baseline [15].

In the present study, the PBO/GMB group showed a rapid improvement in all efficacy endpoints following their transition to open-label galcanezumab. This result is in-line with the findings from the double-blind periods of the PERSIST and other phase 3 trials [13, 15, 16]. Further noticeable improvements in most efficacy endpoints were observed from the end of the double-blind period through to the end of the OLE for patients who received galcanezumab throughout the study. This suggests that patients may achieve continued improvements with galcanezumab treatment for up to 6 months and suggests that longer treatment with galcanezumab may result in greater treatment benefits. However, the interpretability of these data is limited by the lack of a placebo comparator during the OLE, with patients being aware that they were receiving active treatment.

Galcanezumab also had a maintained response during this OLE study, with two-thirds of patients (66.2%) who achieved a clinically meaningful ≥ 50% response during the double-blind phase of the trial maintaining this level of response during the OLE. This suggests patients achieving a good initial response to galcanezumab are likely to continue to show a good response for up to 6 months of treatment.

Galcanezumab previously showed acceptable tolerability during the double-blind phase of the PERSIST trial and this continued throughout longer-term treatment in the OLE. No patients experienced a treatment-related SAE and only three patients (0.6%) discontinued treatment due to a TEAE during the OLE. Furthermore, treatment compliance was high (> 97%) during the OLE. Based on the high treatment adherence and acceptable tolerability observed in the PERSIST study it is likely that patient adherence would be high in a clinical setting, this has important clinical implications, as patients with migraine typically exhibit poor adherence to standard preventive treatments [5]. These data were comparable to the long-term safety profile of galcanezumab reported in previous phase 3 studies and their OLEs [10–15]. The long-term benefits of targeting the CGRP pathway have also been shown in studies of other monoclonal antibodies that target CGRP, including erenumab and fremanezumab [21, 22].

Limitations of this study included that, as for all OLE studies, there was no comparator arm or blinding, thus limiting the interpretability of results from open-label period. Furthermore, the study was only six months in duration, and the longer-term safety and efficacy of galcanezumab in Asian patients remain unknown.

## Conclusions

Once-monthly galcanezumab 120 mg was efficacious and well-tolerated for up to 6 months in patients from China, India, and Russia with episodic migraine. Our results support the long-term findings from OLEs of prior pivotal phase 3 studies of galcanezumab in patients with migraine [13, 15].

### Supplementary Information

**Additional file 1.**

## Data Availability

Lilly provides access to all individual participant data collected during the trial, after anonymization, except for pharmacokinetic or genetic data. Data are available upon reasonable request. Access is provided after a proposal has been approved by an independent review committee identified for this purpose and after receipt of a signed data-sharing agreement. Data and documents, including the study protocol, statistical analysis plan, clinical study report, and blank or annotated case report forms, will be provided in a secure data-sharing environment. For details on submitting a request, see the instructions provided at www.vivli.org.

## Abbreviations

ADA: Antidrug antibody
CGRP: Calcitonin gene-related peptide
ePRO: Electronic patient-reported outcomes
ICHD: International Classification of Headache Disorders
LS: Least squares
MHDs: Migraine headache days
MIDAS: Migraine Disability Assessment
MMRM: Mixed model repeated measures
MSQ: Migraine-Specific Quality of Life Questionnaire
OLE: Open-label extension
PGI-S: Patient Global Impression of Severity
SAE: Serious adverse event
SD: Standard deviation
TEAE: Treatment-emergent adverse event
