# Supplementary material for: Galcanezumab in patients with episodic migraine: results from the open-label period of the phase 3 PERSIST study
Source: J Headache Pain. 2023 Aug 4;24(1):103. doi: 10.1186/s10194-023-01613-1 (PMC10401806; doi:10.1186/s10194-023-01613-1)
Supplement: Supplementary file 1 — Additional file 1. [file 10194_2023_1613_MOESM1_ESM.docx]

**Supplementary Figure 1.** Study design


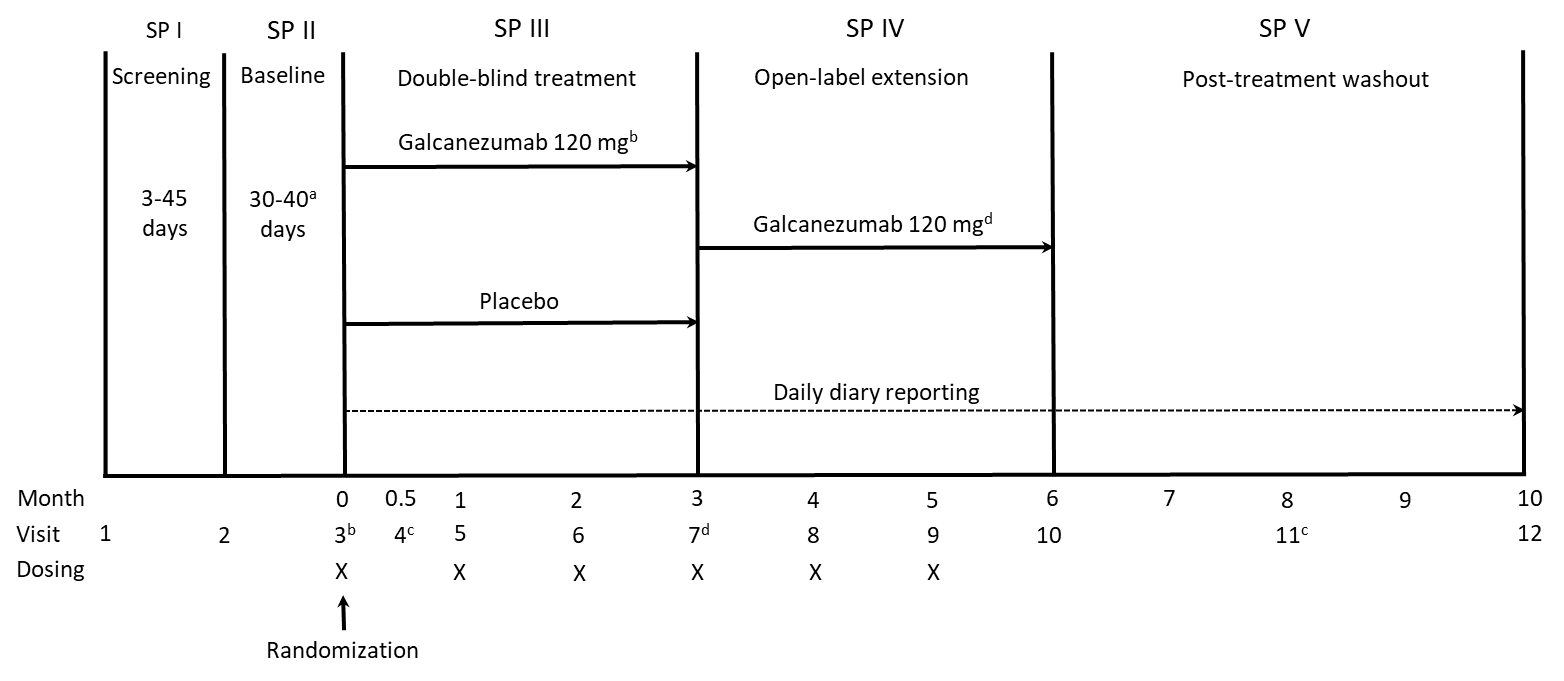


^a^Eligibility period determined between a minimum of 30 days and a maximum of 40 days.

^b^Patients randomized to galcanezumab 120 mg received a loading dose of 240 mg at the first injection only (Visit 3)

^c^Telephone visits

^d^At Visit 7, patients randomized to placebo who entered the open-label extension received galcanezumab at a loading dose of 240 mg, while patients randomized to galcanezumab 120 mg continued treatment at the 120 mg dose.

SP, study period; X indicates when dosing occurred.
